# Supplementary material for: Efficient molecular evolution to generate enantioselective enzymes using a dual-channel microfluidic droplet screening platform
Source: Nat Commun. 2018 Mar 12;9:1030. doi: 10.1038/s41467-018-03492-6 (PMC5847605; doi:10.1038/s41467-018-03492-6)
Supplement: Supplementary file 1 — Supplementary Information [file 41467_2018_3492_MOESM1_ESM.pdf]

Supplementary Information

Efficient molecular evolution to generate enantioselective enzymes using a dual-channel microfluidic droplet screening platform

Fuqiang Ma[a*], Meng Ting Chung[b*], Yuan Yao[c], Robert Nidetz[b], Lap Man Lee[b], Allen P. Liu[bd], Yan Feng[a], Katsuo Kurabayashi[be**], Guang-Yu Yang[a**]

[a]State Key Laboratory of Microbial Metabolism, School of Life Sciences and Biotechnology, Shanghai Jiao Tong University, 800 Dongchuan Road, Shanghai, 200240, China.
[b]Department of Mechanical Engineering, University of Michigan, Ann Arbor, MI, 48109, USA.
[c]MIIT Key Laboratory of Critical Materials Technology for New Energy Conversion and Storage, School of Chemistry and Chemical Engineering, Harbin Institute of Technology, Harbin 150001, China.
[d]Department of Biomedical Engineering, University of Michigan, Ann Arbor, MI, 48109, USA.
[e]Department of Electrical Engineering and Computer Science, University of Michigan, Ann Arbor, MI, 48109, USA.
[*]The first two authors contributed equally to this article
[**]To whom correspondence should be addressed. E–mail: yanggy@sjtu.edu.cn, katsuo@umich.edu

Supplementary Figures

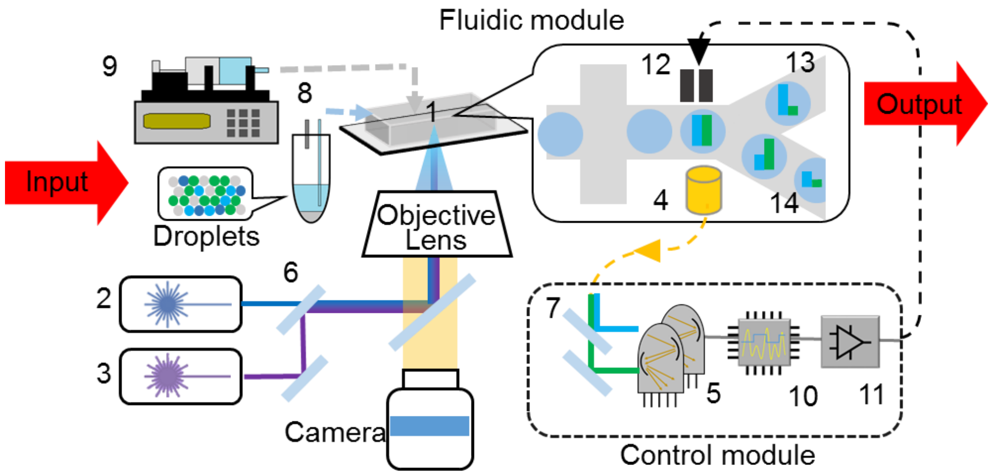

Supplementary Figure 1. Schematic of the experimental setup of the DMDS platform. The detection/sorting device (1) is placed on an inverted microscope (TS-100, Nikon) for observation using a high-speed camera (phantom Miro eX4). Two lasers (2,3) (λ=405 nm, 4 mW and λ=473 nm, 4 mW) are guided through embedded optical fibers (4) (F-MCB-T-1FC, Newport corp.) to excite 7-Hydroxycoumarin-3-carboxylic acid (HCCA) and fluorescein. The detection system incorporates two photomultiplier tubes (5) (Hamamatsu H9306-03) that receive emission signals passing through optical band pass filters (450 nm and 525 nm CWL). Two dichroic mirrors (6,7) (LP425nm and LP505nm) are used to combine and separate different excitation wavelengths. Optical emission signals are detected and converted to voltage signals by the PMTs, and are recorded by a DAQ card (PCI-6111, National Instrument). A stable pressure source provided by a pressure regulator (MPR1-1, Dwyer) is used to draw water-in-oil droplets from a centrifuge tube (8) into the detection and sorting device. The spacer oil phase (pure Novec7500) is pumped into the flow channel from a syringe (9) (10 mL, BD) using a syringe pump (KDS-200, KD scientific). The control module is composed of a customized signal processing circuit and a micro controller board (10) (Uno32, Chipkit) to generate TTL signals. The TTL signals trigger a function generator (33220A, Agilent) to generate square wave pulses which are subsequently amplified through a voltage amplifier (11) (AV-110B-PS-D, Avtech) and sent to the microelectrodes (12) of the detection and sorting device. Only droplets emitting signals detected above a threshold readout are deflected to the collection outlet (13) and the rest of the droplets flow to the waste outlet (14).

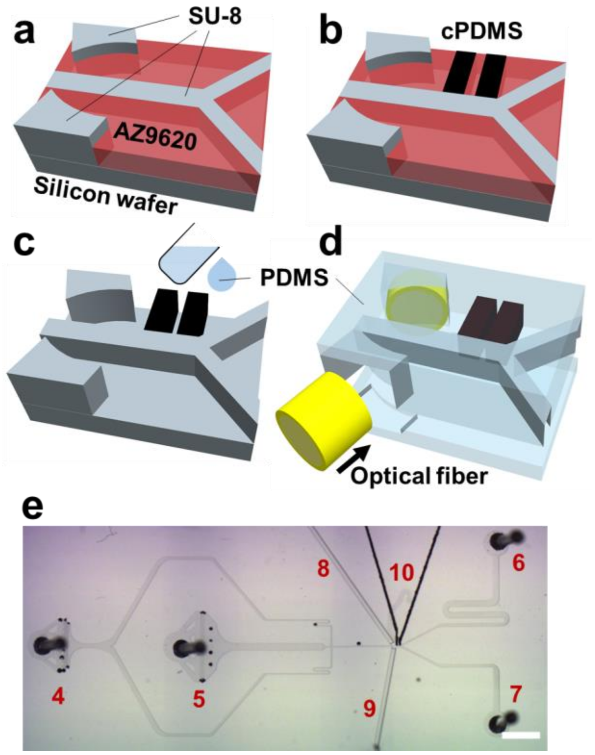

Supplementary Figure 2. Steps in the process of the DC-FADS device fabrication. **a** An SU8 mold was first fabricated on a Si wafer by standard photolithography, followed by spin coating of a 40 μm thick AZ 9620 layer to cover the uncovered bare Si surface. **b** Cavities were formed within the AZ 9620 by photolithography and then manually filled with a carbon-doped PDMS precursor (25% w/w). The carbon-doped PDMS was cured, and the AZ 9620 layer was removed using a photoresist developer solution. **c** A PDMS precursor was poured onto the mold. After curing at 60 ^{o}C overnight, the PDMS layer was peeled off from mold together with the carbon-doped PDMS structures, which formed sorting electrodes near the PDMS flow channel wall. **d** The PDMS layer was bonded to another PDMS layer, resulting in a whole device structure with the features of fiber channels, flow channels, and electrodes. Optical fibers were inserted into the fiber channels, which allowed them to be mechanically aligned with the main flow channel at a fixed angle. **e** Photography of finished device. **4** spacer oil phase inlet; **5** water-in-oil emulsion droplet reinjection inlet; **6** sorting collection channel outlet; **7** waste channel outlet; **8** excitation optical fiber; **9** detection optical fiber; **10** sorting electrodes. Scale bar: 2 mm.

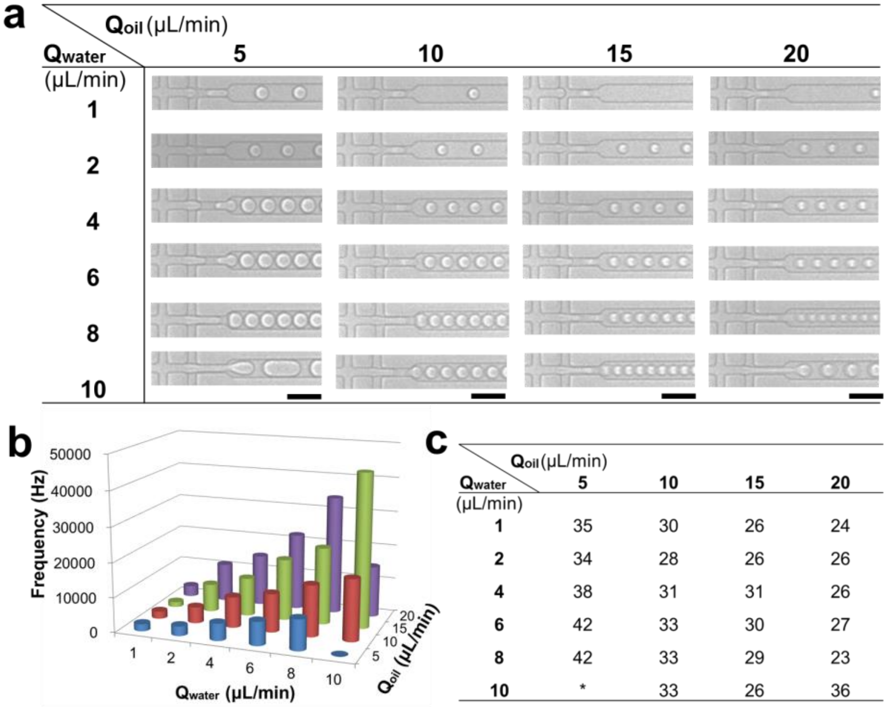

Qwater (μL/min) \ Qoil (μL/min)	5	10	15	20
**1**	35	30	26	24
**2**	34	28	26	26
**4**	38	31	31	26
**6**	42	33	30	27
**8**	42	33	29	23
**10**	*	33	26	36

Supplementary Figure 3. Droplet generation under different liquid flow rates. **a** Optical images, **b** throughput, and **c** diameter (μm) of generated droplets at different flow rates of aqueous and oil phases (Q_{water} and Q_{oil}). *: unstable droplet generation. A fluorinated oil, Novec7500 (3M), containing 2% (w/w) 008 fluorinate surfactant (RAN Biotechnologies) was used as the oil phase. The FFDG device adopted the design of Florian Hollfelder's group[1, 2], as optimized for the current study. Scale bars: 100 μm.

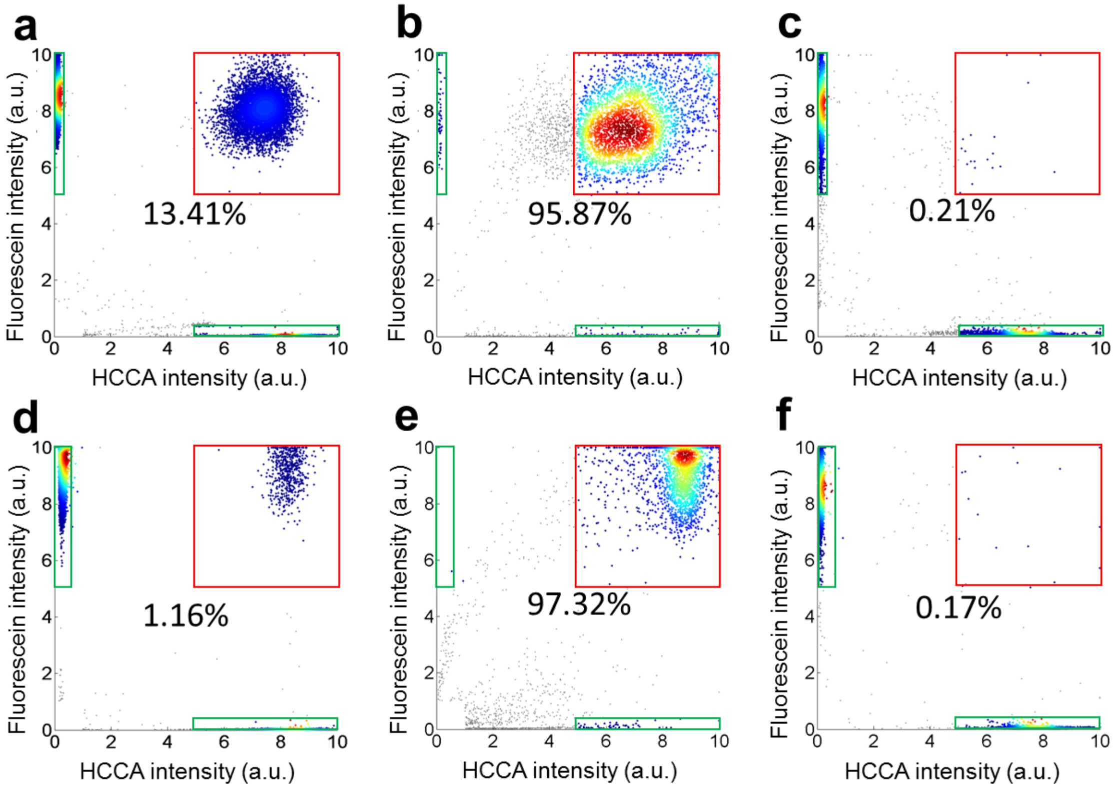

Supplementary Figure 4. Results of double-color based droplet sorting. **a** Initial droplet population distribution prior to sorting. The dot plots in the square indicate that the HCCA + fluorescein double-color droplets (the target droplets for the sorting) occupied 13.4% of the population in the mixture. **b** Droplet population distribution in the collection channel after sorting. The double-color droplets were found for 95.9% of the sorted droplets. **c** Droplet population distribution in the waste channel after sorting. The double-color droplets were found for only 0.21 % of the droplets in the waste. **d** Pre-sorting droplet population distribution in a mixture with a double-color droplet population fraction of 1.2%. **e** Post-sorting droplet population distribution in the collection channel with a double-color droplet population fraction of 97.3%. **f** Post-sorting droplet population distribution in the waste channel with a double-color droplet population fraction of 0.17%. For each enrichment assay, ~10000 positive droplets were sorted and re-injected for purity evaluation.

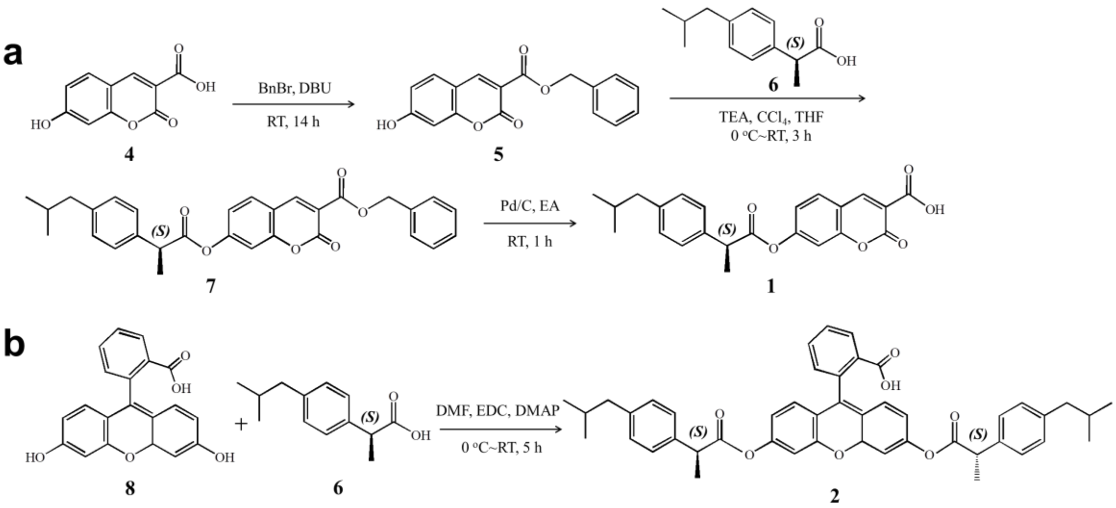

Supplementary Figure 5. Synthesis of fluorogenic substrates derived from ibuprofen. **a** synthesis of substrate **1**, **b** synthesis of substrate **2**. Synthesis of substrate **3** was the same as that of substrate **2**. Since fluorophores in droplet-based assays must exhibit good retention behavior, we chose fluorescein and 7-Hydroxycoumarin-3-carboxylic acid (HCCA) as the substrate-appended fluorophores in our experiments. Fluorescein has been widely used in droplet-based assays with a moderate retention time in droplets.[3, 4] HCCA is a carboxyl group modified coumarin derivative, which is significantly improved in retention behavior (more than 95% of fluorescence can be retained within 30-min of off-chip incubation, data not shown) and thus suitable for use in droplet-based assays.[5, 6]

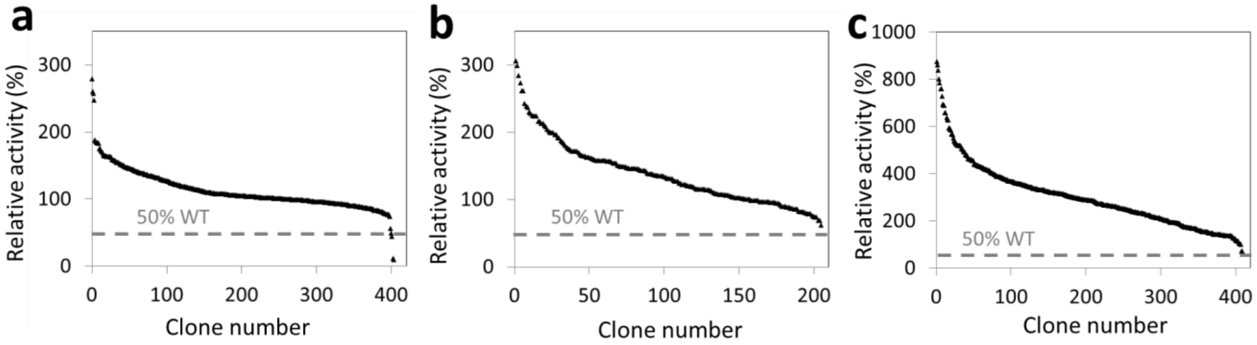

Supplementary Figure 6. The proportions of active variants after DMDS screening. **a** Mutants enriched from Library 1, with 99% of the population showing activity for *(S)*-ibuprofen. **b** Mutants enriched from Library 2, with 100% of the population showing activity for *(S)*-ibuprofen. **c** Mutants enriched from Library 3, with 100% of the population showing activity for *(S)*-ibuprofen. The active variants were identified as mutants with activities higher than 50% of wild-type AFEST.

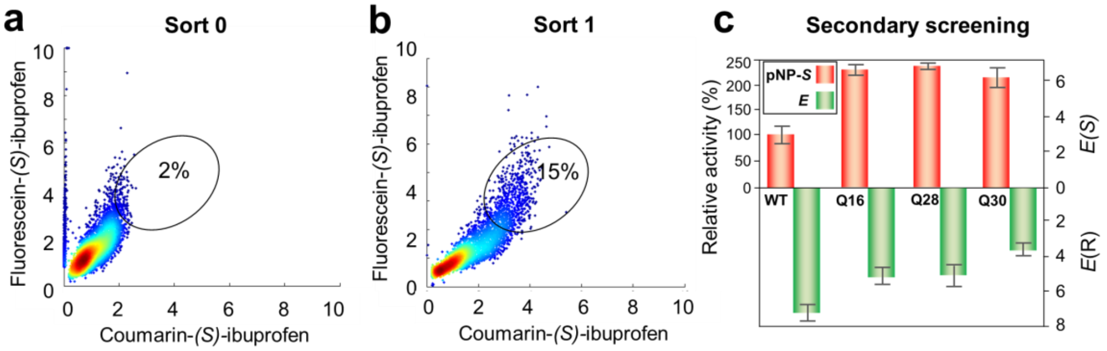

d

Mutant	Mutation modes
Q16	Pro8Leu, Tyr11Asn, Ile209Val, Asp211Gly, Ser216Gly
Q28	Pro8Leu, Tyr11Asn, Asn37Ser, Asp211Gly, Ser216Gly, Asn302Ser
Q30	Ile209Val, Asp211Gly, Leu257Pro

Supplementary Figure 7. Screening of the DNA shuffling library (Library 2). The mutants were isolated from Library 1 employing substrate **1** and **2** in the cooperative mode. **a** Activity distribution of the original DNA shuffling library towards substrate **1** and substrate **2**. 2% of the most active mutants toward both substrates were sorted. **b** Activity distribution of mutants after one round of enrichment towards both substrates. The percentage in the sorting gate was enriched to 15%. After a secondary screening based on 96-well plates assays, positive mutants with improved activity as well as enantioselectivity towards *(S)*-ibuprofen-*p*NP ester were identified **c** and their mutation sites were shown in **d**. In **c**, the red bar represents activity (left axis) and the green bar represents enantioselectivity (right axis). Values are mean ± SD of triplicate samples in an experiment. Experiments were independently repeated three times (**c**).

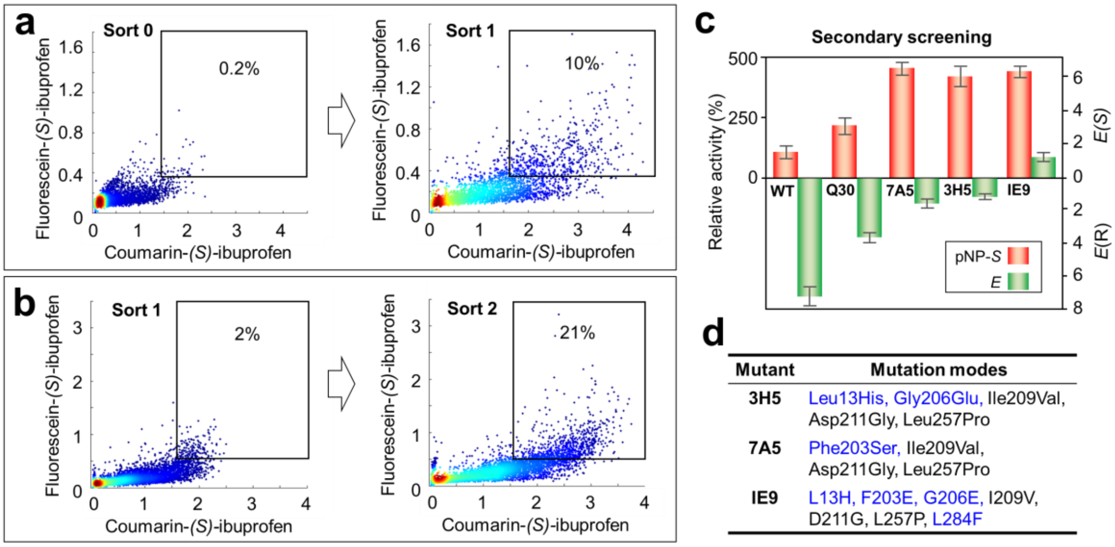

Supplementary Figure 8. Screening of Library 3 in the cooperative mode. **a** Enrichment effectiveness of the 1st round of sorting. The percentage of mutants in the sorting gate increased from 0.2% to 10% after the 1st round of sorting. **b** Enrichment effectiveness of the 2nd round of sorting. The percentage of mutants in the sorting gate increased from 2% to 21% after the 2nd round of sorting. Positive mutants with further improved activity as well as enantioselectivity toward *(S)*-ibuprofen-*p*NP ester are shown in **c** and their mutations are shown in **d** (the newly identified mutations are shown in blue). In **c**, the red bar represents activity (left axis) and the green bar represents enantioselectivity (right axis). Note: different PMT detection voltages were applied in each round of screening with the purpose of clearly distinguishing mutants with different properties. Therefore, the difference between Supplementary Figure 6 and Supplementary Figure 7 in activity distribution toward substrate **1** and **2** is caused by different PMT detection voltages applied, instead of by the altered substrate selectivity. Values are mean ± SD of triplicate samples in an experiment. Experiments were independently repeated three times (**c**).

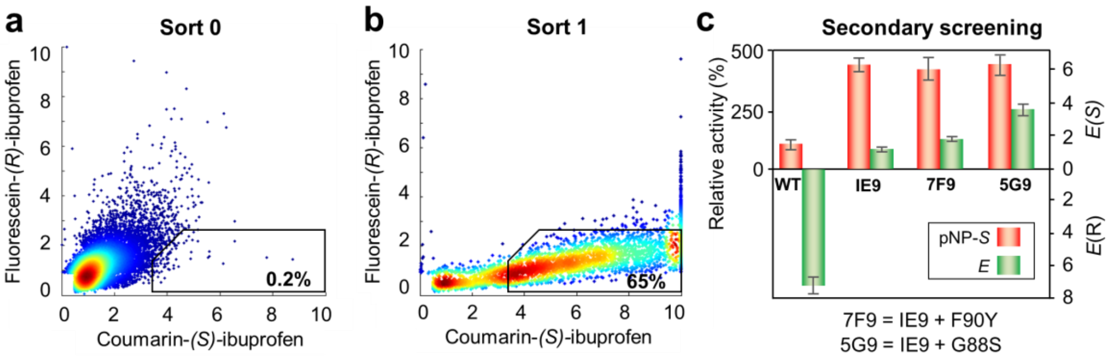

Supplementary Figure 9. Screening of Library 4 in the biased mode. **a** Activity distribution of the original library towards substrate **1** and substrate **3**. 0.2% of the mutants with high activity toward substrate **1** but low activity toward substrate **3** were sorted. **b** Activity distribution of mutants after one round of enrichment. The percentage in the sorting gate was enriched to 65%. After a secondary screening based on 96-well plates assays, two positive mutants with improved enantioselectivity towards *(S)*-ibuprofen-*p*NP ester were identified and shown in **c**. In **c**, the red bar represents activity (left axis) and the green bar represents enantioselectivity (right axis). Values are mean ± SD of triplicate samples in an experiment. Experiments were independently repeated three times (**c**).

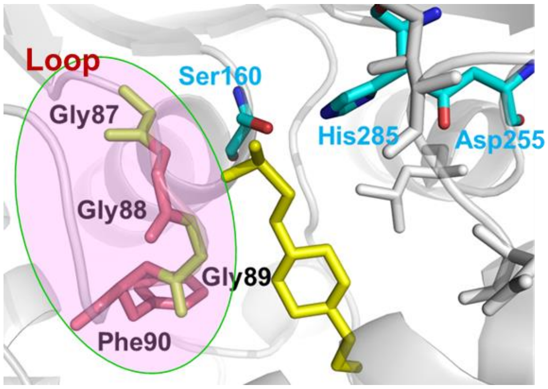

Supplementary Figure 10. Position of 88 and 90 sites in crystal structure of AFEST. These two sites belong to Loop87-90, which is adjacent to the substrate. The residues in the catalytic triad are shown in cyan.

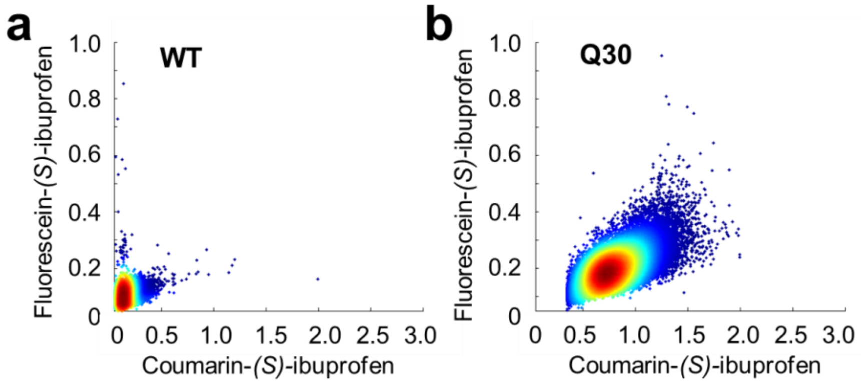

Supplementary Figure 11. Activity of WT AFEST **a** and Q30 **b** in cooperative mode. Q30 showed significantly improved activities toward both coumarin-*(S)*-ibuprofen (substrate **1**) and fluorescein-*(S)*-ibuprofen (substrate **2**). WT AFEST and Q30 were detected under the same PMT voltages.

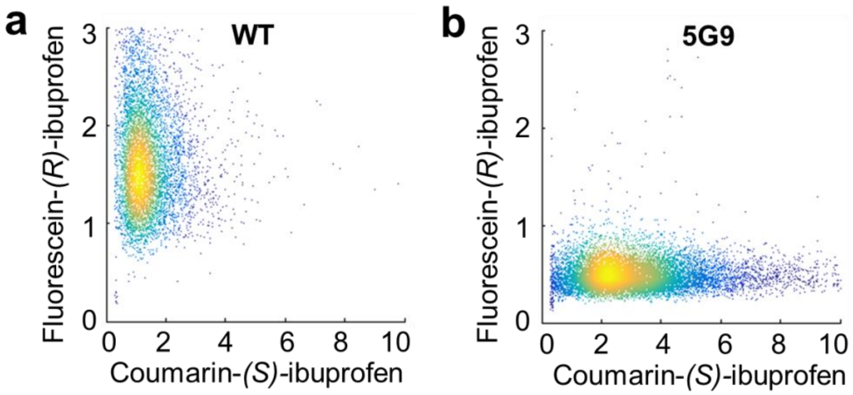

Supplementary Figure 12. Enantioselectivity of WT AFEST **a** and 5G9 **b** in biased mode. Compared with wild-type AFEST, the 5G9 mutant showed significantly increased activities toward coumarin-(*S*)-ibuprofen (substrate **1**) but decreased activity toward fluorescein-(*R*)-ibuprofen (substrate **3**), which was in accordance with its improved enantioselectivity toward (*S*)-ibuprofen substrates. WT AFEST and 5G9 were detected under the same PMT voltages.

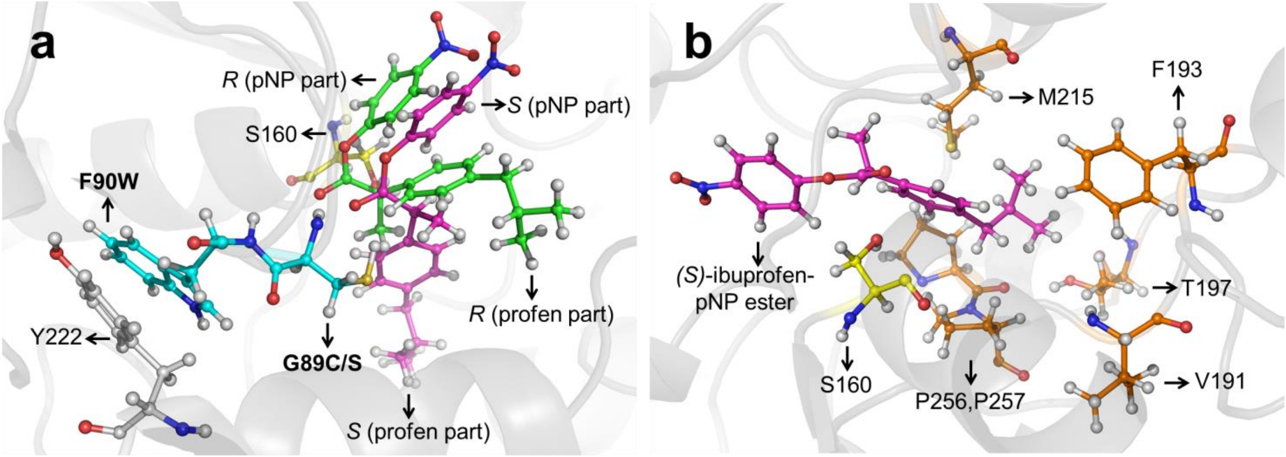

Supplementary Figure 13. Mechanism of 6A8 and 4D11 with distinct enantioselectivity. **a** Docking poses of *(R)*/*(S)*-ibuprofen-*p*-nitrophenol ester with 6A8 and 4D11. Mutation G89C of 6A8 introduced significant steric hindrance against *(R)*-ibuprofen-*p*-nitrophenol ester (the shortest distance between the –SH group of the mutation and the substrate was 0.83 Å), but no obvious steric hindrance against *(S)*-ibuprofen-*p*-nitrophenol ester (the shortest distance was 2.2 Å). Similar steric hindrance was also introduced by the G89S mutation of 4D11. Further, the F90W mutation of 6A8 enhanced the pi-pi interaction with Y222, which stabilized the oxyanion hole structure (G88, G89, A166) yet meanwhile decreased the flexibility of the substrate pocket. **b** The profen moiety of the *(R)*-ibuprofen-*p*-nitrophenol ester is surrounded by hydrophobic residues (in orange), forming hydrophobic interactions.

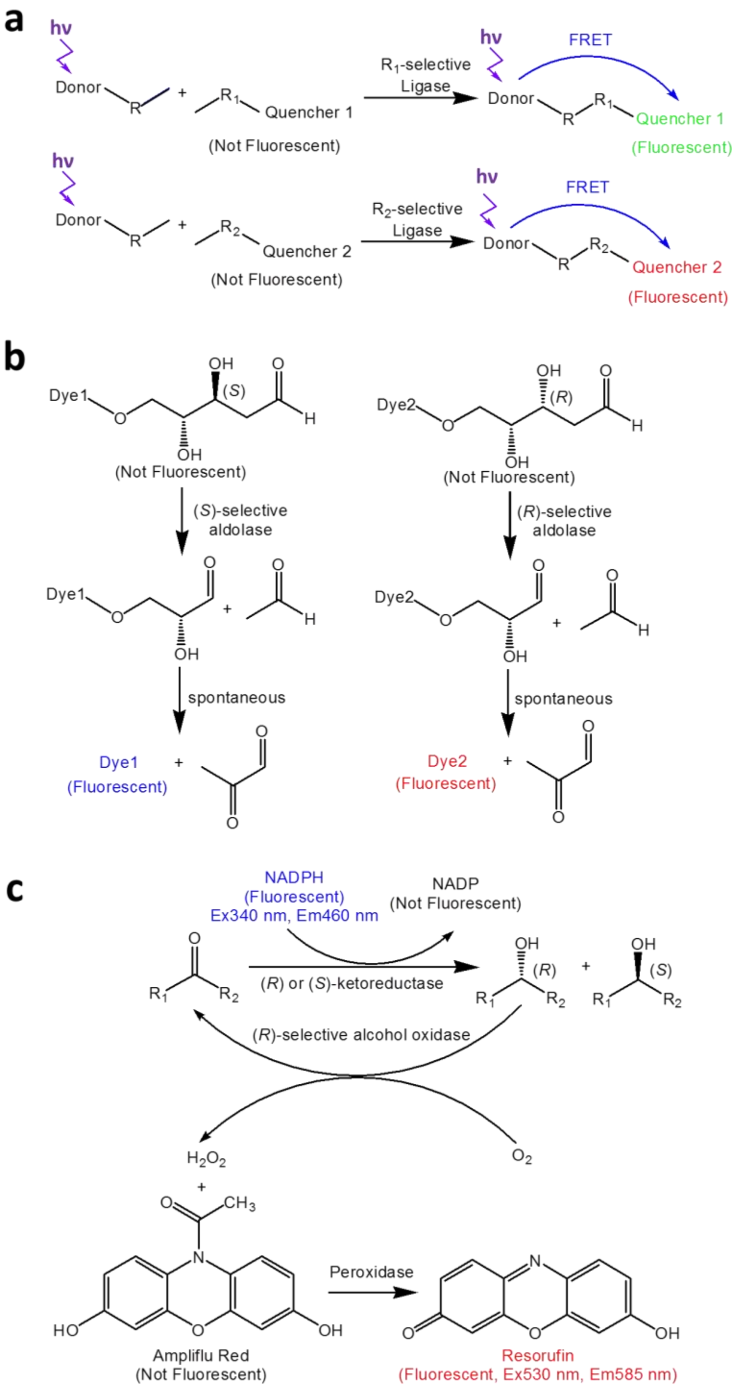

Supplementary Figure 14. Dual-florescence assays for screening enzymatic selectivity. **a** A strategy for screening selectivity of ligases. **b** A strategy for screening enantioselectivity of aldolases. The dye moieties may be coumarin or resorufin derivatives. **c** A strategy for screening enantioselectivity of ketoreductases. It is noteworthy that NADPH can be oxidized by the commonly used horseradish peroxidase (HRP). To solve this problem, evolved HRP or other peroxidase species that are inactive toward NADPH would need to be developed.

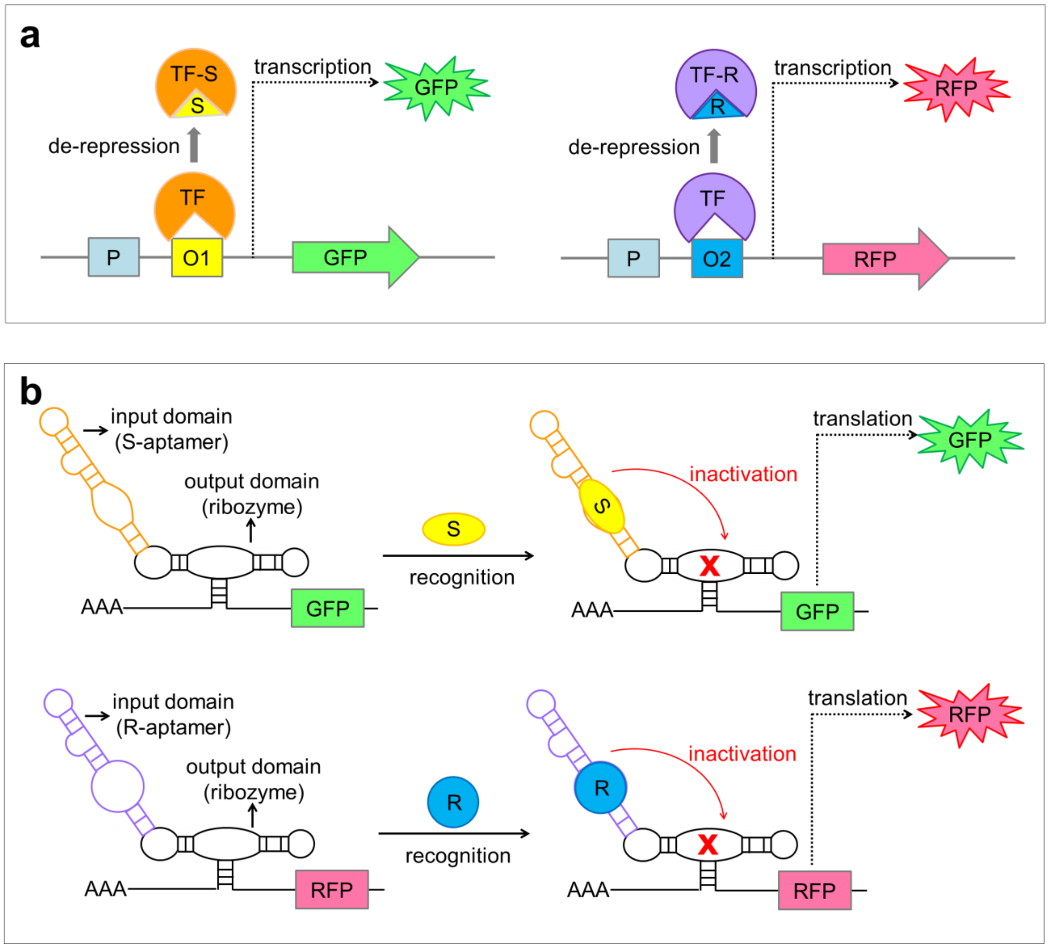

Supplementary Figure 15. Biosensor-based strategies for screening enantioselectivity. **a** A strategy that employs enantiomer-responsive transcriptional factors, **b** a strategy that employs riboswitches.

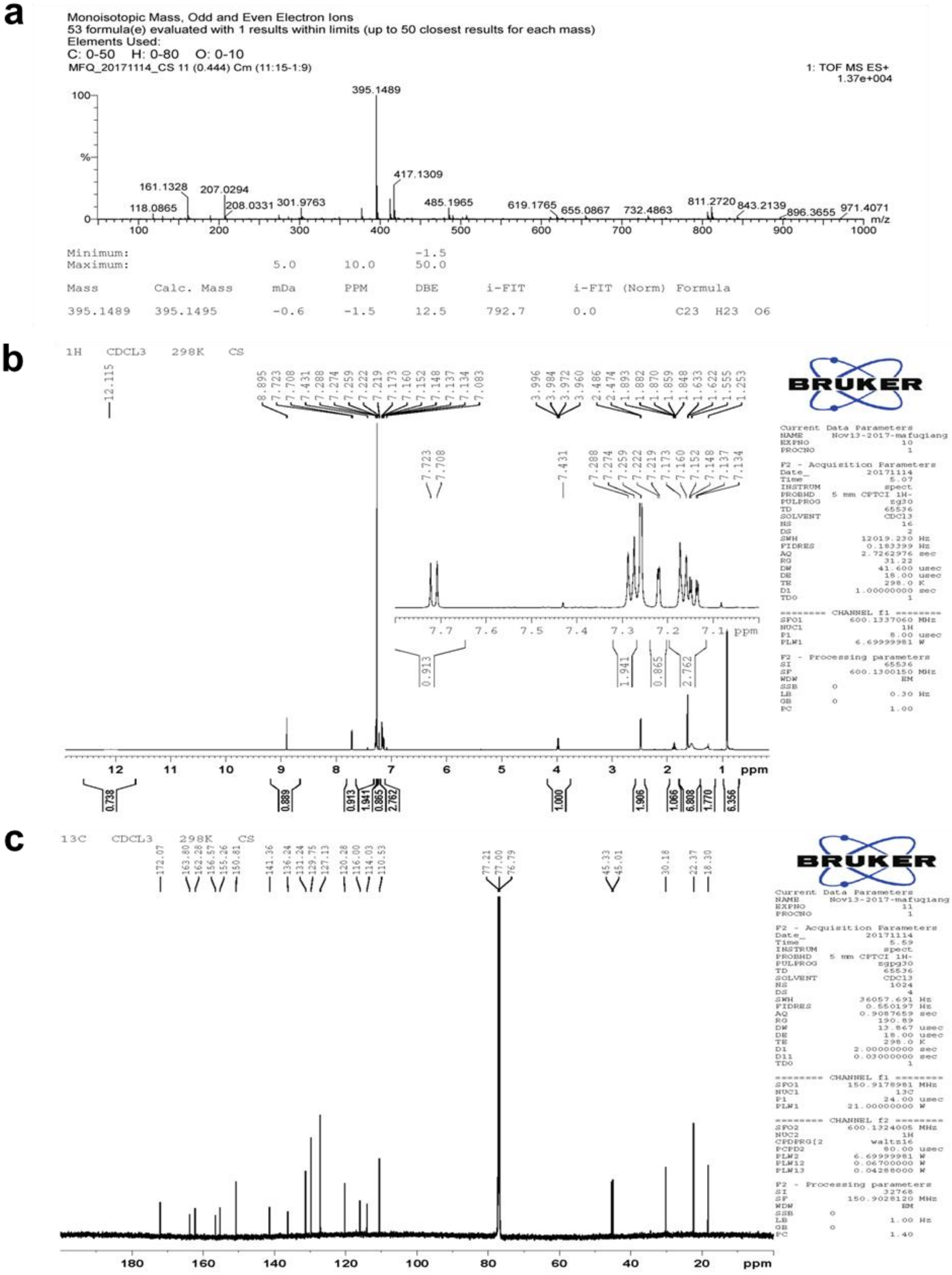

Supplementary Figure 16. Characterization of substrate **1**. **a** mass spectrum, **b** ^{1}H NMR spectrum, **c** ^{13}C NMR spectrum.

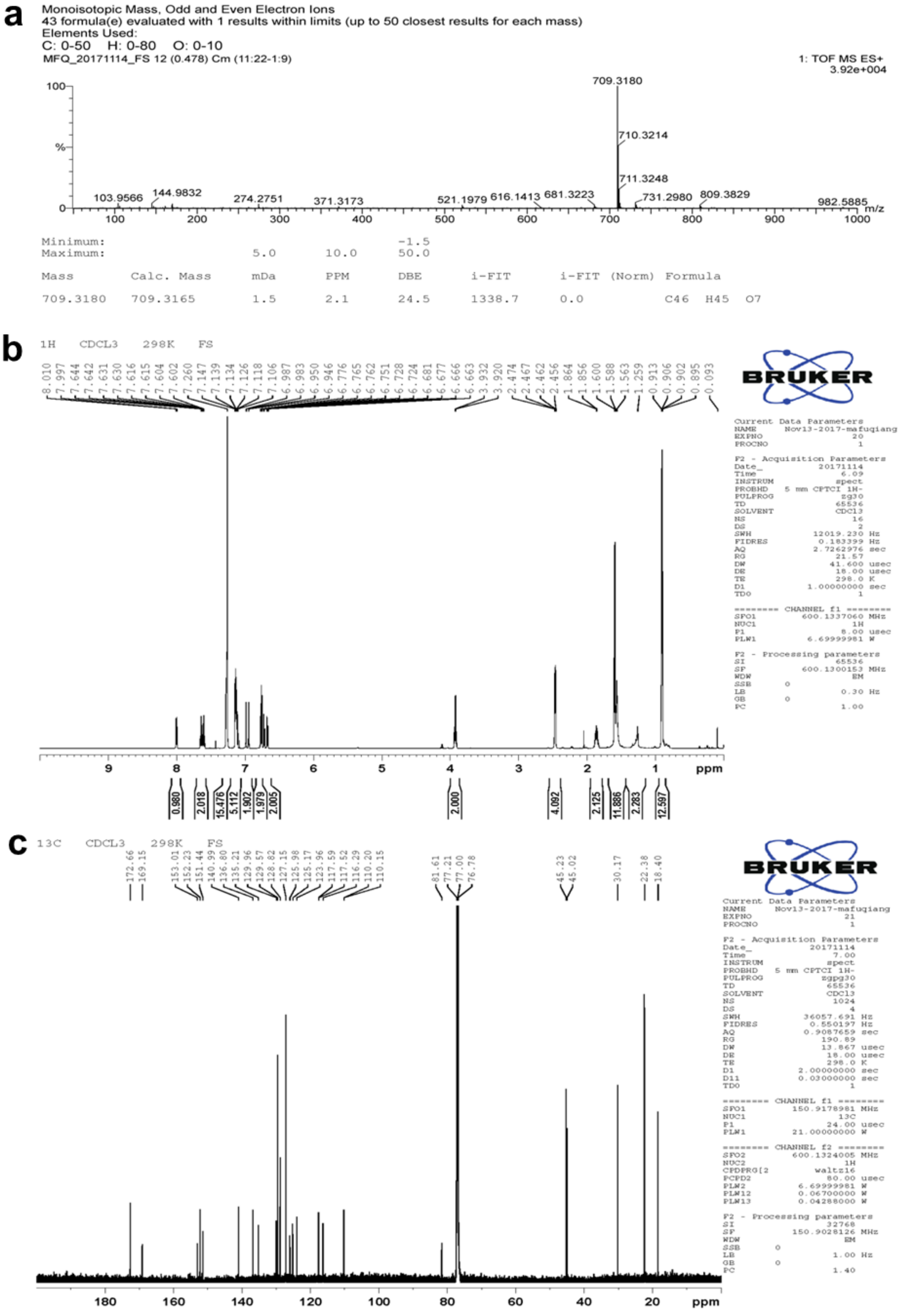

Supplementary Figure 17. Characterization of substrate **2**. **a** mass spectrum, **b** ^{1}H NMR spectrum, **c** ^{13}C NMR spectrum.

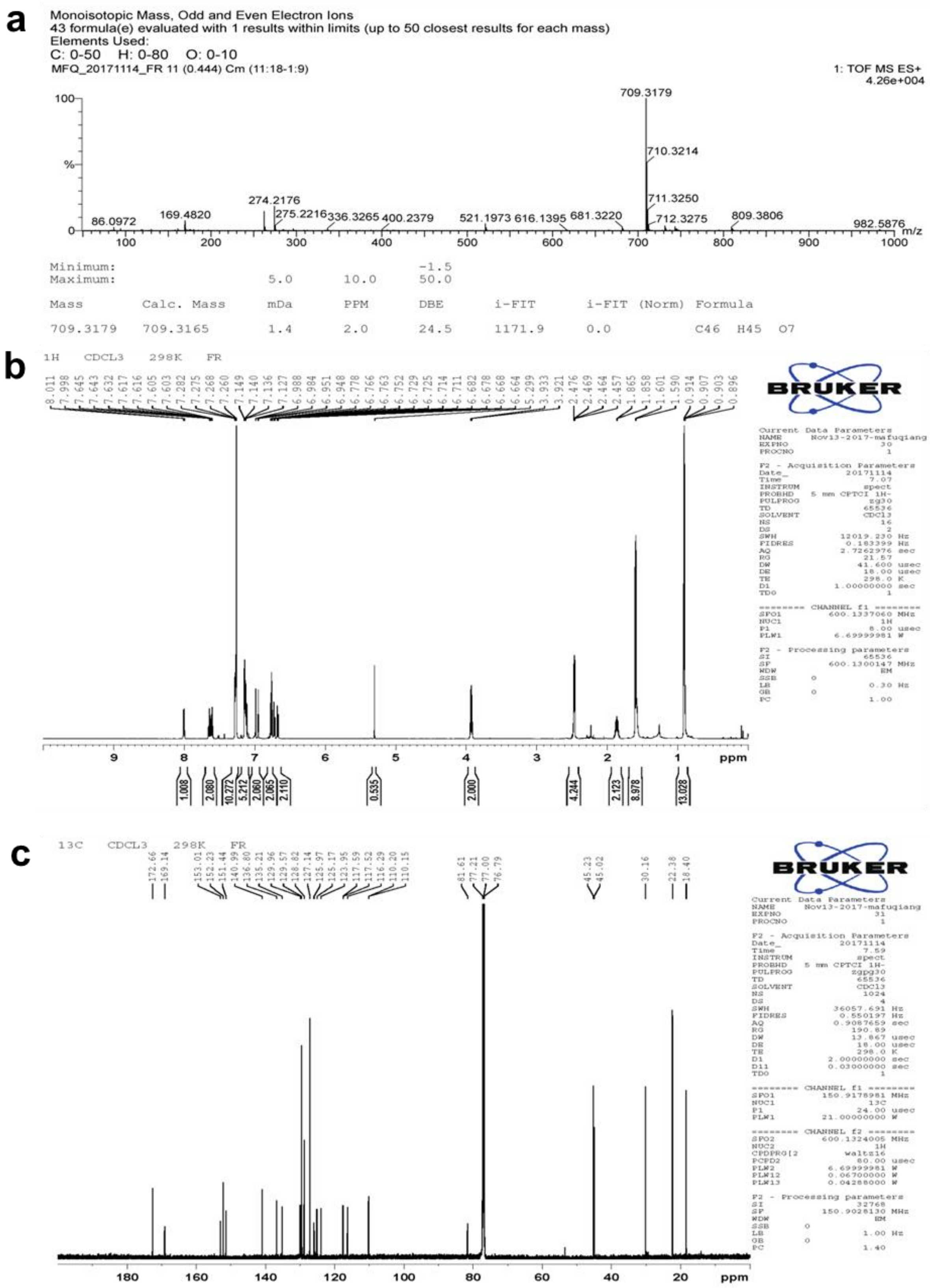

Supplementary Figure 18. Characterization of substrate **3**. **a** mass spectrum, **b** ^{1}H NMR spectrum, **c** ^{13}C NMR spectrum.

Supplementary Tables

Supplementary Table 1. Summary of the whole screening process.

Library number	Library type	Template	Library size	DMDS Screening mode	Number of variants for secondary screening	Proportion of active variants[b]
**1**	Ep-PCR[a]	WT AFEST	2,000,000	cooperative	400	99%
**2**	DNA shuffling	7 mutants from Library 1	500,000	cooperative	200	100%
**3**	Ep-PCR	Q30	2,000,000	cooperative	400	100%
**4**	Ep-PCR	IE9	500,000	biased	200	n.d.[c]
**5**	site-saturation mutagenesis	IE9	10,000	biased	100	n.d.[c]

[a] Ep-PCR: Error-prone PCR

[b] The proportion of active variants was identified as the ratio of mutants which showed activities >50% of the wild-type AFEST.

[c] n.d. Not determined. This is because Library 4 and Library 5 were screened based on the enantioselectivity change, so some of the variants that had higher enantioselectivity but overall lower enzyme reaction activities and were included in the secondary screening.

Supplementary Table 2. Summary of positive mutants with improved activity toward (*S*)-ibuprofen-*p*-nitrophenol (*p*NP) ester identified from Library 1.

Mutant	Relative activity (%)	Mutation modes
**WT**	100	None
**T1**	137	Asn37Ser
**T2**	157	Asn37Ser, Ile209Val, Asp211Gly, Asn302Ser
**T3**	136	Ser216Gly
**T4**	174	Pro8Leu, Tyr11Asn, Glu41Lys, Ile209Val, Asp211Gly, Asn302Ser
**T5**	209	Pro8Leu, Tyr11Asn, Glu41Lys, Ile209Val, Asp211Gly
**T6**	189	Asp211Gly, Leu257Pro
**T7**	171	Leu257Pro

Supplementary Table 3. Summary of positive mutants identified from the site-saturation mutagenesis libraries targeting residues on Loop87-90 (Library 5).

Mutant	*E*	Mutations on IE9	Library identified from
IE9	1.1 *(S)*		Library 3
7F9	1.6 *(S)*	F90Y	Library 4
5G9	3.6 *(S)*	G88S	Library 4
6A8	>100 *(S)*	G89C, F90W	Library 5
4D11	80 *(S)*	G89S	Library 5
1C8	60 *(S)*	G89C	Library 5
6D6	14 *(S)*	G89Y	Library 5
5C5	13 *(S)*	G89S, F90W	Library 5
3H10	8.2 *(S)*	G88R	Library 5
3B11	2.0 *(S)*	G87S, G88R	Library 5
1D4	1.9 *(S)*	G87S	Library 5
4E12	74 *(R)*	G89P	4th site-saturation
4C1	17 *(R)*	G89P, F90L	4th site-saturation

Supplementary Table 4. Mutation sites of key mutants throughout the evolution process of AFEST.

Enzyme	Mutation sites
**WT**	wild type
**Q30**	I209V, D211G, L257P
**IE9**	L13H, F203E, G206E, I209V, D211G, L257P, L284F
**7A5**	L13H, F90Y, F203E, G206E, I209V, D211G, L257P, L284F
**5G9**	L13H, G88S, F203E, G206E, I209V, D211G, L257P, L284F
**6A8**	L13H, G89C, F90W, F203E, G206E, I209V, D211G, L257P, L284F
**4D11**	L13H, G89S, F203E, G206E, I209V, D211G, L257P, L284F
**4E12**	L13H, G89P, F203E, G206E, I209V, D211G, L257P, L284F

Supplementary Table 5. The sequences of all primers used in this study.

Primer name	Sequence (5'-3')
AEFST-F	GGTACTCCTCTAGAGATGCTTGATATGCCAATCG (underlined: *Xba*I site)
AEFST-R	CCAAAACAGAAGCTTCTAGTCGAACACAAGAAGAGC (underlined: *Hind* III site)
WHOP-87-F	CTGGTTTACTATCACNNKGGTGGATTTGTGATTTGCAGCATCG
WHOP-87-R	GCAAATCACAAATCCACCMNNGTGATAGTAAACCAGAACC
WHOP-88-F	CTGGTTTACTATCACGGTNNKGGATTTGTGATTTGCAGCATCG
WHOP-88-R	GCAAATCACAAATCCMNNACCGTGATAGTAAACCAGAACC
WHOP-89-F	GGTTTACTATCACGGTGGTNNKTTTGTGATTTGCAGCATCGAGTCG
WHOP-89-R	CGATGCTGCAAATCACAAAMNNACCACCGTGATAGTAAACCAGAACC
WHOP-90-F	GGTTTACTATCACGGTGGTGGANNKGTGATTTGCAGCATCGAGTCG
WHOP-90-R	CGATGCTGCAAATCACMNNTCCACCACCGTGATAGTAAACCAGAACC
WHOP-87/88-F	CTGGTTTACTATCACNNKNNKGGATTTGTGATTTGCAGCATCG
WHOP-87/88-R	GCAAATCACAAATCCMNNMNNGTGATAGTAAACCAGAACC
WHOP-87/89-F	CTGGTTTACTATCACNNKGGTNNKTTTGTGATTTGCAGCATCG
WHOP-87/89-R	GCAAATCACAAAMNNACCMNNGTGATAGTAAACCAGAACC
WHOP-87/90-F	CTGGTTTACTATCACNNKGGTGGANNKGTGATTTGCAGCATCG
WHOP-87/90-R	GCAAATCACMNNTCCACCMNNGTGATAGTAAACCAGAACC
WHOP-88/89-F	CTGGTTTACTATCACGGTNNKNNKTTTGTGATTTGCAGCATCG
WHOP-88/89-R	GCAAATCACAAAMNNMNNACCGTGATAGTAAACCAGAACC
WHOP-88/90-F	CTGGTTTACTATCACGGTNNKGGANNKGTGATTTGCAGCATCG
WHOP-88/90-R	GCAAATCACMNNTCCMNNACCGTGATAGTAAACCAGAACC
WHOP-89/90-F	GGTTTACTATCACGGTGGTNNKNNKGTGATTTGCAGCATCGAGTCG
WHOP-89/90-R	CGATGCTGCAAATCACMNNMNNACCACCGTGATAGTAAACCAGAACC

Note: AFEST-F and AFEST-R are used to clone the genes of *afest* and its variants into pUC18 vector. Primers labeled with WHOP are used for creating site-saturation mutagenesis libraries.

Supplementary Discussion

Discussion regarding the versatility of the DMDS system

The DMDS system reported in this paper provides a platform for the detection and sorting of two fluorescent signals simultaneously in micro-droplets at a speed of more than one million reads per hour. However, it is equally important to develop fluorogenic assays for the enzymatic reactions of interest. In recent years, a large variety of fluorogenic enzymatic assays have been developed for the high throughput screening of enzymes of all six of the major EC catalytic categories.[7, 8] Besides the fluorogenic surrogate substrates that we used in this study, other strategies include fluorescence resonance energy transfer (FRET),[9] enzyme-coupled assays,[10] functional group selective reagents,[11] transcriptional factors,[12] riboswitches[13] and nano-sensors.[14] Most of these assays can (in theory) be used in the DMDS system with very simple modifications, thus opening up the possibility for ultrahigh-throughput screening for a great many enzymatic reactions.

For instance, current technology allows the screening of both ***ligases*** and ***lyases*** using DMDS. For example, a FRET-based assay has been developed for DNA ligases[15] and for Ubiquitin ligases.[16] As illustrated in Supplementary Figure 14a (below), it is possible to link two discrete substrate fragments to separate moieties (e.g., a FRET donor and FRET quencher). Different FRET signals then can be used to screen for the substrate specificities for ligases.

Fluorescence assays have also been developed for ***lyases***. Taking aldolases as an example, the fluorogenic aldol substrates have been designed such that it can be catalyzed by the β-elimination activity of aldolases to give 3-oxopropyl umbelliferyl ether, which is subsequently converted into umbelliferone via a spontaneous *β*-elimination reaction.[17, 18] As illustrated in Supplementary Figure 14b, two fluorogenic substrates can be designed by using two aldol enantiomers that are conjugated to different fluorophores. After enzymatic reaction, substrate selectivity can be evaluated by measuring the fluorescence ratios between two fluorophores (similar with the case of hydrolases).

A similar strategy has been used for the screening of C-C bond cleavage ***transferases*** such as transaldolases[19] and transketolase.[20]

Note that the DMDS system can be also used for the screening of ketoreductases, an important class of ***oxidoreductase*** enzyme. Truppo *et al.* (2008) developed a fluorescent assay for the enantioselectivity of ketoreductases in microtiter plates.[21] This strategy can be directly used in the screening for the enantioselectivity of ketoreductases in our DMDS system. As illustrated in Supplementary Figure 14c, the (*R*)-alcohol product could be detected specifically by coupling an (*R*)-selective alcohol oxidase, generating resorufin with red fluorescence. Meanwhile, the consumption of NADPH which represents the total production of (*R*)- and (*S*)-alcohol products is detected by measuring the decrease of NADPH fluorescence.[22, 23] The enantioselectivity of the ketoreductase can be calculated as the ratio between coumarin fluorescence and decreased NADPH fluorescence.

Furthermore, with the recent fast development of biosensors (such as transcriptional factors or riboswitches), it is possible to convert the concentration of any targeted compound into the overexpression of downstream reporter (e.g. fluorescence proteins).[24] As illustrated in Supplementary Figure 15, it is possible to design artificial biosensors for any enzymatic products of interest to activate various reporter genes, which will greatly promote the application of DMDS system.[25-27]

Moving forward, ever more complicated enzymatic properties can be screened using the DMDS system by adding further hardware or chemical technologies. For example, by fusing a target enzyme with a fluorescence protein, the enzymatic activity and expression level can be detected simultaneously, which allows the directly measuring of specific activity. Also, the DMDS system can in theory be used to detect the activities of more than one key enzyme in a metabolic pathway, which would be much more informative than the currently-typical detection of a single end product. Above all, we believe that the DMDS system can broaden the application of droplet-based ultrahigh throughput screening.

Supplementary Methods

Synthesis of HCCA-*(S)*-ibuprofen ester (substrate 1).

7-Hydroxycoumarin-3-carboxylic acid (**4** in Supplementary Figure 5a) (HCCA) was synthesized according to the protocol reported by Singh *et al.*[7] The ^{1}H NMR (200 MHz, DMSO-d_6) results were: δ 8.68 (s, 1H), 7.75 (d, *J*=8.4 Hz, 1H), 6.85 (d, *J*=8.8 Hz, 1H), and 6.74 (s, 1H).

Benzyl 7-hydroxy-2-oxo-2H-chromene-3-carboxylate (**5** in Supplementary Figure 5a) was synthesized as an intermediate product. To a mixture of HCCA (700 mg, 3.4 mmol) and DBU (568 mg, 3.74 mmol) in DMF (20 mL), (bromomethyl) benzene (635 mg, 3.74 mmol) was added slowly at room temperature. The mixture was stirred at room temperature for 14 h. TLC (EtOAc:PE=1:1) measurement was taken to confirm the completion of the reaction. The reaction solution was poured into water (100 mL) and filtered to extract compound **5** (720 mg, yield: 72%), forming a crude yellow solid, which was used in the next step without further purification.

Benzyl 7-({2-[4-(2-methylpropyl)phenyl]propanoyl}oxy)-2-oxo-2H-chromene-3-carboxylate (**7** in Supplementary Figure 5a) was synthesized as another intermediate product. To a solution of compound **5** (720 mg, 2.43 mmol) in CCl_4 (15 mL) and THF (15 mL), TEA (358 mg, 3.55 mmol) was added slowly at 0 ^{o}C, then (*S*)-ibuprofen (**6**) (618 mg, 3 mmol) was added. The mixture was warmed to room temperature and stirred for 3 h. After TLC (EtOAc : PE = 1:1) measurement, the reaction was quenched with water, and the reaction system was extracted by adding DCM to the solution, dried and concentrated. The residue was purified by silica gel column chromatography (EtOAc:PE=1:3 to 1:1) to obtain compound **7** in Supplementary Figure 5b (820 mg, yield: 69.7%) forming a light yellow oil.

7-({2-[4-(2-methylpropyl)phenyl]propanoyl}oxy)-2-oxo-2H-chromene-3-carboxylic acid (**1**) was synthesized as a final product. To a solution of compound **7** (484 mg, 1 mmol) in EtOAc (20 mL), Pd/C (85 mg, 15%) was added. The mixture was stirred under H_2 (50 Psi) for 1 h. TLC (EtOAc:PE=1:1) measurement was taken to confirm the completion of the reaction. The reaction system was then filtered, the solvent was removed, and the residue was crystallized from EtOAc:PE=1:5 to obtain compound **1** (HCCA-(*S*)-profen) forming a pale yellow solid (208 mg, yield: 52.8%). ^{1}H-NMR ($CDCl_3$, 400 MHz) results were δ: 0.92 (d, *J* = 4.0 Hz, 6H), 1.63 (d, *J* = 8.0 Hz 3H), 1.86-1.88 (m, 1H), 2.48 (d, *J* = 4.0 Hz, 2H), 3.98 (q, *J* = 8.0 Hz 1H), 7.16-7.18 (m, 3H), 7.22 (d, *J* = 1.6 Hz, 1H), 7.28 (d, *J* = 4.0 Hz, 2H), 7.71 (d, *J* = 4.0 Hz, 1H) and 8.90 (s, 1H). ^{13}C-NMR($CDCl_3$, **100** MHz) δ: 18.33, 22.40, 30.21, 45.04, 45.36, 110.56, 114.05, 116.02, 120.31, 127.16, 129.77, 131.27, 136.26, 141.39, 150.84, 155.28, 156.59, 162.31, 163.83 and 172.10. HRMS (m/z): $[M]^+$ calcd. for $C_{23}H_{23}O_6$, 395.1495; found, 395.1489.

Synthesis of fluorescein-*(S)*-ibuprofen ester (substrate 2).

(1-ethyl-3-(3-dimethylaminopropyl)carbodiimide hydrochloride) (EDC) (372 mg, 2.4 mmol) and 4-

dimethylaminopyridine (DMAP) (12.2 mg, 0.1 mmol) were added slowly to a solution of fluorescein (**8** in Supplementary Figure 5b) (Sigma-Aldrich) (332 mg, 1.0 mmol) in DMF (20 mL) at 0 ^{o}C, then (*S*)-ibuprofen (**6** in Supplementary Figure 5b) (495 mg, 2.4 mmol) was added to the solution. The mixture was warmed to room temperature and stirred for 5 h. TLC (EtOAc : PE=1:1) measurement was taken to confirm the completion of the reaction. The reaction system was extracted by adding dichloromethane (DCM) to the solution, dried, and concentrated. The residue was purified by silica gel column chromatography (EtOAc:PE = 1:4 to 1:1) to obtain fluorescein-(*S*)-ibuprofen (380 mg, yield: 53.4%) forming a pale yellow solid. ^{1}H-NMR ($CDCl_3$, 400 MHz) results were δ: 0.91 (d, *J* = 4.0 Hz, 12H), 1.60 (d, *J* = 4.0 Hz, 6H), 1.85-1.87 (m, 2H), 2.46-2.48 (m, 4H), 3.92 (q, *J* = 8.0 Hz 1H), 6.67 (dd, *J* = 8.0, 1.6 Hz, 2H), 6.77 (d, *J* = 4.0 Hz, 2H), 6.97 (d, *J* = 16.0 Hz, 2H), 7.13-7.15 (m, 5H), 7.26-7.30 (m, 4H), 7.60-7.65 (m, 2H), and 8.01 (d, *J* = 8.0 Hz, 1H). ^{13}C-NMR($CDCl_3$, 100 MHz) δ: 18.43, 22.41, 30.20, 45.05, 45.25, 81.64, 110.18, 110.23, 116.31, 117.56, 117.62, 123.99, 125.20, 126.01, 127.18, 128.85, 129.60, 129.99, 135.24, 136.83, 141.02, 151.47, 152.26, 153.04, 169.18 and 172.69. HRMS (m/z): $[M]^+$ calcd. for $C_{46}H_{45}O_7$, 709.3165; found, 709.3180

Synthesis of fluorescein-*(R)*-ibuprofen ester (substrate 3).

The synthesis of substrate **3** is similar with the synthesis of substrates **2**. ^{1}H-NMR ($CDCl_3$, 400 MHz) results were δ: 0.91 (d, *J* = 4.0 Hz, 12H), 1.60 (d, *J* = 4.0 Hz, 6H), 1.85-1.87 (m, 2H), 2.46-2.48 (m, 4H), 3.92 (q, *J* = 8.0 Hz 1H), 6.67 (dd, *J* = 8.0, 1.6 Hz, 2H), 6.77 (d, *J* = 4.0 Hz, 2H), 6.97 (d, *J* = 16.0 Hz, 2H), 7.13-7.15 (m, 5H), 7.26-7.30 (m, 4H), 7.60-7.65 (m, 2H), and 8.01 (d, *J* = 8.0 Hz, 1H). ^{13}C-NMR($CDCl_3$, **100** MHz) δ: 18.43, 22.41, 30.20, 45.05, 45.25, 81.64, 110.18, 110.23, 116.31, 117.56, 117.62, 123.99, 125.20, 126.01, 127.18, 128.85, 129.60, 129.99, 135.24, 136.83, 141.02, 151.47, 152.26, 153.04, 169.18 and 172.69. HRMS (m/z): $[M]^+$ calcd. for $C_{46}H_{45}O_7$, 709.3165; found, 709.3179.

Supplementary References

1. Fischlechner, M. et al. Evolution of enzyme catalysts caged in biomimetic gel-shell beads. *Nat. Chem.* **6**, 791-796 (2014).
2. Zinchenko, A. et al. One in a Million: Flow cytometric sorting of single cell-lysate assays in monodisperse picolitre double emulsion droplets for directed evolution. *Anal. Chem.* **86**, 2526-2533 (2014).
3. Baret, J. C. et al. Fluorescence-activated droplet sorting (FADS): efficient microfluidic cell sorting based on enzymatic activity. *Lab Chip* **9**, 1850-1858 (2009).
4. Colin, P. Y. et al. Ultrahigh-throughput discovery of promiscuous enzymes by picodroplet functional metagenomics. *Nat. Commun.* **6**, 10008 (2015).
5. Ma, F. et al. Substrate Engineering Enabling Fluorescence Droplet entrapment for IVC-FACS based ultrahigh-throughput screening. *Anal. Chem.* **88**, 8587-8595 (2016).
6. Woronoff, G. et al. New generation of amino coumarin methyl sulfonate-based fluorogenic substrates for amidase assays in droplet-based microfluidic applications. *Anal. Chem.* **83**, 2852-2857 (2011).
7. Reymond, J. L., Fluxà, V. S. & Maillard, N. Enzyme assays. *Chem. Commun. (Camb)* **1**, 34-46 (2009).
8. Goddard, J. P. & Reymond, J. L. Recent advances in enzyme assays. *Trends Biotechnol.* **22**, 363-370 (2004).
9. Yang, G. et al. Fluorescence activated cell sorting as a general ultra-high-throughput screening method for directed evolution of glycosyltransferases. *J. Am. Chem. Soc.* **132**, 10570-10577 (2010).
10. Kumagai, K., Kojima, H., Okabe, T. & Nagano, T. Development of a highly sensitive, high-throughput assay for glycosyltransferases using enzyme-coupled fluorescence detection. *Anal. Biochem.* **447**, 146-155 (2014).
11. Gotor, R., Ashokkumar, P., Hecht, M., Keil, K. & Rurack, K. Optical pH sensor covering the range from pH 0-14 compatible with mobile-device readout and based on a set of rationally designed indicator dyes. *Anal. Chem.* **89**, 8437-8444 (2017).
12. Mahr, R. & Frunzke, J. Transcription factor-based biosensors in biotechnology: current state and future prospects. *Appl. Microbiol. Biotechnol.* **100**, 79-90 (2016).
13. Su, Y., Hickey, S. F., Keyser, S. G. L. & Ming, C. H. *In vitro* and *in vivo* enzyme activity screening via RNA-based fluorescent biosensors for S-adenosyl-l-homocysteine (SAH). *J. Am. Chem. Soc.* **138**, 7040-7047 (2016).
14. Zhang, L., Zhao, J., Zhang, H., Jiang, J. & Yu, R. Double strand DNA-templated copper nanoparticle as a novel fluorescence indicator for label-free detection of polynucleotide kinase activity. *Biosens. Bioelectron.* **44**, 6-9 (2013).
15. Shapiro, A. B., Eakin, A. E., Walkup, G. K. & Rivin, O. A high-throughput fluorescence resonance energy transfer-based assay for DNA ligase. *J. Biomol. Screen.* **16**, 486-493 (2011).
16. Sun, Y. Overview of approaches for screening for ubiquitin ligase inhibitors. *Methods Enzymol.* **399**, 654-663 (2005).
17. Jourdain, N., Carlón, R. P. & Reymond, J. L. A stereoselective fluorogenic assay for aldolases: detection of an anti-selective aldolase catalytic antibody. *Tetrahedron Lett.* **39**, 9415-9418 (1998).
18. Pérez, C. R., Jourdain, N. & Reymond, J. L. Fluorogenic polypropionate fragments for detecting stereoselective aldolases. *Chemistry* **6**, 4154 -4162 (2000).
19. Gonzálezgarcía, E. et al. Fluorogenic stereochemical probes for transaldolases. *Chemistry* **9**, 893-899 (2003).
20. Sevestre, A., Hélaine, V., Guyot, G., Martin, C. & Hecquet, L. A fluorogenic assay for transketolase from Saccharomyces cerevisiae. *Tetrahedron Lett.* **44**, 827-830 (2003).
21. Truppo, M. D., Escalettes, F. & Turner, N. J. Rapid determination of both the activity and enantioselectivity of ketoreductases. *Angew. Chem. Int. Ed. Engl.* **47**, 2639-2641 (2008).
22. Blacker, T. S. et al. Separating NADH and NADPH fluorescence in live cells and tissues using FLIM. *Nat. Commun.* **5**, 3936 (2014).
23. Patterson, G. H., Knobel, S. M., Arkhammar, P., Thastrup, O. & Piston, D. W. Separation of the glucose-stimulated cytoplasmic and mitochondrial NAD(P)H responses in pancreatic islet beta cells. *Proc. Natl. Acad. Sci. U S A* **97**, 5203-5207 (2000).
24. Eggeling, L., Bott, M. & Marienhagen, J. Novel screening methods--biosensors. *Curr. Opin. Biotechnol.* **35**, 30-36 (2015).
25. Looger, L. L., Dwyer, M. A., Smith, J. J. & Hellinga, H. W. Computational design of receptor and sensor proteins with novel functions. *Nature* **423**, 185-190 (2003).

26. Michener, J. K. & Smolke, C. D. High-throughput enzyme evolution in *Saccharomyces cerevisiae* using a synthetic RNA switch. *Metab. Eng.***14**, 306-316 (2012).
27. Wang, M., Li, S. & Zhao, H. Design and engineering of intracellular-metabolite-sensing/regulation gene circuits in Saccharomyces cerevisiae. *Biotechnol. Bioeng.* **113**, 206-215 (2016).
